# Women hold up half the sky – and half the burden of the HIV epidemic

**DOI:** 10.7448/IAS.16.1.18608

**Published:** 2013-03-08

**Authors:** Shirin Heidari, Susan Kippax, Papa Salif Sow, Mark A Wainberg

**Affiliations:** 1 Journal of the International AIDS Society, Geneva, Switzerland; 2 Social Policy Research Centre, University of New South Wales, Sydney, Australia; 3 Department of Infectious Diseases, University of Dakar, Dakar, Senegal; 4 McGill University AIDS Centre, Jewish General Hospital, Montreal, QC, Canada

## Abstract

It has been said that women hold up half the sky. In the HIV epidemic, women carry half the burden of the epidemic, perhaps even more. The HIV burden on women is dramatically higher in some regions, certain age groups and among marginalized groups, such as female sex workers. Women's vulnerability to HIV is exacerbated by gender inequality and domestic violence.

The global effort towards elimination of paediatric HIV and keeping mothers alive deserves applause. However, the needs of women go beyond their child-bearing age or potentials and/or reproductive desires and must be recognized in the global HIV agenda. In particular, more female-controlled prevention tools are urgently required to allow women to protect themselves.

It is time to turn the tide through promoting gender equality and genuinely committing to gender-responsive policies and programmes, and encouraging a more gender-aware research agenda that can generate necessary evidence. In recognition of International Women's Day, the *Journal of the International AIDS Society* is pleased to launch a thematic series to highlight articles that address the different dimensions of HIV as they relate to women.

It has been said that women hold up half the sky [1]. In the HIV epidemic, women carry half the burden of the epidemic, perhaps even more. Although HIV initially was more prevalent among men, the burden of disease shifted quickly and HIV incidence among women has equalled that of men. By the end of 2011, almost exactly half, 49%, of people living with HIV were women, according to the latest UNAIDS figures [2, 3].

The HIV burden on women is dramatically higher in some regions: in sub-Saharan Africa and the Caribbean nearly 60% of people living with HIV are women. In certain age groups, women are also at greater risk of HIV infection: adolescent girls in sub-Saharan Africa constitute 71% of all youth aged 15 to 24 living with HIV [2, 3]. Furthermore, among married discordant couples, women remain at greater risk for acquisition of HIV than do men. In west Africa, for example, 25% of new infections occur in married heterosexual women. Women among certain marginalized groups are worst affected; sex workers are 13.5 times more likely to be infected with HIV than other women; and in some studies, women who inject drugs have been reported to have a 50% higher HIV prevalence than men who inject drugs [3]. A particular sub-population of women that are especially absent in the context of HIV are lesbian, bisexual, queer and transsexual (LBQT) women, who are frequently subject to violence, stigma and discrimination, putting them at higher HIV risk. HIV prevalence among transgender women, who may have consensual or non-consensual sex with men, has been reported to range from 11.8 to 27.7% [2, 4].

In general, women's vulnerability to HIV acquisition is exacerbated by gender inequality and domestic violence. Addressing gender-based violence in national HIV programmes has been proven to reduce the risk of HIV transmission, encourage HIV disclosure and improve adherence to antiretroviral therapy (ART) [5, 6]. More female-controlled prevention tools that do not require male consent or participation are vital if we are to make progress in the years to come.

It would be simplistic to measure the burden of the epidemic only in terms of epidemiological data. Women carry a disproportionately large burden as primary caregivers for HIV-positive family members and also shoulder most of the unpaid household work. More equitable caregiving responsibility between men and women is a fundamental, though often forgotten, aspect of the global response. As a featured theme of the on-going 57th Commission on the Status of Women, a renewed effort to urge actions for an equal sharing of responsibilities in the context of HIV caregiving between men and women is therefore welcome [7].

The global HIV community has taken steps towards addressing the changing face and the feminization of the epidemic, with some noticeable success. Owing to an accelerated effort in line with the Global Plan towards the elimination of new HIV infections among children by 2015 and keeping their mothers alive, the proportion of pregnant women living with HIV and receiving antiretroviral therapy in the 21 high priority countries in sub-Saharan Africa indentified in the Global Plan jumped from 16% in 2009 to 48% in 2011 [8]. These figures may account for the higher ART coverage rates observed in women as compared with men in low- and middle-income countries (68% vs. 47%, respectively) [2]. Better ART coverage among women than men is likely due to underlying factors, such as the different health-seeking behaviours of women and men, and the fact that women have multiple entry points to care, including antenatal clinics and prevention of mother to child transmission programmes.

The needs of women, however, go beyond their child-bearing age or potentials and/or reproductive desires and must be recognized in the global HIV agenda. According to the UNAIDS 2012 country progress report, of the total HIV funding allocated for women in 2009 to 2011, 71% was invested in prevention of vertical transmission, and only 5% was allocated to prevention of gender-based violence [3, 9].

The concept of “know your epidemic” is an important mantra that has been rightfully advocated, though it has not been extended fully to women. Data on HIV prevalence and treatment coverage reported by countries do provide a general overview of the HIV status in men and women. However, details with respect to different sub-populations are frequently absent, and data are seldom disaggregated by sex or age. Structural barriers apply to women regardless of age and sexual preference, and are influenced by complex gender dynamics and intertwined with socio-economic factors that reduce women's ability to protect themselves from HIV infection. Gender dimension must be integrated into programmes and accounted for across the continuum of care.

In recognition of International Women's Day, the *Journal of the International AIDS Society* is pleased to launch a thematic series to highlight articles that address the different dimensions of HIV as they relate to women. With this launch, we are also welcoming new contributions.

In an article by Rujumba *et al*., published in this thematic series, the authors provide insight into such factors as stigma, fear of intimate partner violence and loss of financial support in case of abandonment as barriers to HIV disclosure by HIV-positive pregnant women to their partners [10]. To better respond to the realities of women, Carter *et al*. provide an overview of what is considered to be female-specific programmes and propose a conceptual definition of women-centred programming in the context of HIV [11]. A qualitative study of LBQT women by Logie *et al*. sheds light on the complex challenges faced by LBQT women, underlining the negligence of the HIV risk for this population, translating into an absence of directed efforts [4].

Other relevant contributions are underway, and we welcome additional submissions that can fill the outstanding gaps on issues pertaining to women living with, at risk of or affected by HIV across ages and sub-populations.

More than 30 years into the epidemic, it is time to turn the tide through promoting gender equality and genuinely committing to gender-responsive policies and programmes, and encouraging a more gender-aware research agenda that can generate necessary evidence.
